# Profiling extracellular vesicle release by the filarial nematode *Brugia malayi* reveals sex-specific differences in cargo and a sensitivity to ivermectin

**DOI:** 10.1371/journal.pntd.0006438

**Published:** 2018-04-16

**Authors:** Hiruni Harischandra, Wang Yuan, Hannah J. Loghry, Mostafa Zamanian, Michael J. Kimber

**Affiliations:** 1 Department of Biomedical Sciences, College of Veterinary Medicine, Iowa State University, Ames, Iowa, United States of America; 2 Department of Pathobiological Sciences, School of Veterinary Medicine, University of Wisconsin-Madison, Madison, Wisconsin, United States of America; McGill University, CANADA

## Abstract

The filarial nematode *Brugia malayi* is an etiological agent of Lymphatic Filariasis. The capability of *B*. *malayi* and other parasitic nematodes to modulate host biology is recognized but the mechanisms by which such manipulation occurs are obscure. An emerging paradigm is the release of parasite-derived extracellular vesicles (EV) containing bioactive proteins and small RNA species that allow secretion of parasite effector molecules and their potential trafficking to host tissues. We have previously described EV release from the infectious L3 stage *B*. *malayi* and here we profile vesicle release across all intra-mammalian life cycle stages (microfilariae, L3, L4, adult male and female worms). Nanoparticle Tracking Analysis was used to quantify and size EVs revealing discrete vesicle populations and indicating a secretory process that is conserved across the life cycle. *Brugia* EVs are internalized by murine macrophages with no preference for life stage suggesting a uniform mechanism for effector molecule trafficking. Further, the use of chemical uptake inhibitors suggests all life stage EVs are internalized by phagocytosis. Proteomic profiling of adult male and female EVs using nano-scale LC-MS/MS described quantitative and qualitative differences in the adult EV proteome, helping define the biogenesis of *Brugia* EVs and revealing sexual dimorphic characteristics in immunomodulatory cargo. Finally, ivermectin was found to rapidly inhibit EV release by all *Brugia* life stages. Further this drug effect was also observed in the related filarial nematode, the canine heartworm *Dirofilaria immitis* but not in an ivermectin-unresponsive field isolate of that parasite, highlighting a potential mechanism of action for this drug and suggesting new screening platforms for anti-filarial drug development.

## Author summary

*Brugia malayi* is a parasitic nematode and etiological agent of Lymphatic Filariasis (LF), a mosquito-borne Neglected Tropical Disease affecting approximately 120 million people globally. *Brugia* and other parasitic nematodes have the ability to modulate host biology and evade the immune response but the mechanisms by which they do this are unclear. One possibility is via immunomodulatory proteins and small RNAs packaged and released in extracellular vesicles (EV) that target host cells. Here we show that all *Brugia* life stages relevant to human infection release EVs that are internalized by murine macrophages. These EVs contain immunomodulatory proteins and show sex-specific differences in cargo providing insight into how *B*. *malayi* establishes and maintains infection. Critically, we show ivermectin, a drug used to treat LF and related diseases affects EV release, which provides new insight into how these drugs work and identifies a new screening paradigm to help identify future anti-parasitic drugs.

## Introduction

Lymphatic filariasis (LF) is a Neglected Tropical Disease caused by the parasitic filarial nematodes *Wuchereria bancrofti*, *Brugia malayi*, and *B*. *timori*. Since 2000, the Global Program for the Elimination of Lymphatic Filariasis has orchestrated a concerted elimination effort that is centered on mass drug administration (MDA) augmented with surveillance practices and vector control, and supported by morbidity management and disability prevention strategies. This program has been relatively successful and levels of infection have been reduced in many areas [1] but LF remains a significant global health concern [1,2]. A contributing factor to continued disease transmission is the sub-optimal portfolio of MDA drugs [3]. None of diethylcarbamazine citrate, albendazole or ivermectin are particularly effective at killing adult filarial nematodes [4–6] meaning there is no ‘cure’ for LF and the mechanisms by which these drugs exert their microfilaricidal effects are not resolved.

LF is transmitted through the bite of infected mosquitoes. Infectious L3 stage nematodes access the human host through the puncture wound left by the vector, migrate rapidly to the lymphatic vasculature and molt to the L4 stage, where remarkable growth precedes a further molt to adult worms, which may live for 10–12 years with females releasing microfilariae (mf) larvae for a significant portion of this time. The circulating mf are ingested by vector species during subsequent blood meals, wherein they develop to the infectious L3 stage [7,8].

The immune response to filarial infection is complex and can change during the course of infection. In the broadest terms, a polarized Th2-type response is elicited early in infection and characterized by increased levels of canonical Th2 cytokines, elevated IgG1, IgG4 and IgE, and eosinophilia with macrophages displaying alternative activation phenotypes [9–11]. Chronic infection sees this response modified within an IL-10 regulatory environment that is associated with alternatively activated macrophage phenotypes, reduced T cell proliferation and a general state of immunological tolerance. A final endpoint can be the development of pathological disease featuring increased pro-inflammatory Th1, Th9 and Th17 responses [9,11]. These immunological outcomes are dictated by the balanced interaction between parasite and host and there is considerable evidence that filarial nematodes contribute to these outcomes by secreting protein effectors capable of actively modulating the host immune response [12–21]. What is less clear is our understanding of the mechanistic basis for such parasite-driven manipulations; the pathways for effector molecule release from the worms and delivery to host cells require clarification.

Extracellular vesicles (EV) are membrane-bound vesicles secreted into the extracellular environment by both eukaryotic and prokaryotic cells. The EV population can be heterogeneous and refer to larger microvesicles (150–1,500 nm), formed by budding of the surface plasma membrane, and smaller vesicles (40–150 nm) of endosomal origin, originally termed exosomes [22]. The recognition that exosomes are important mediators of cell-to-cell communication [23,24] has triggered an explosion of interest in EV biology, attributing functions to EV in an array of non-communicable and infectious diseases, whilst recognizing their diagnostic and therapeutic potential [25–28]. The idea that parasitic helminths secrete EV and that these vesicles function at the host parasite interface is an emerging concept with EV release from both nematode [29–33] and platyhelminth species [34–39] recently documented. We have shown that infectious L3 stage *B*. *malayi* release prodigious quantities of discretely sized EVs, of a size and morphology consistent with exosomes [32]. These EV, which were internalized by host immune cells, contain putative effector proteins and microRNA (miRNA) with host identity suggesting multiple avenues by which host biology may be modulated.

Here we profile EV release across the remaining intra-mammalian life stages of *B*. *malayi* (mf, L4, adult male and female). Our results indicate that EV release is not restricted to infective stage parasites but is conserved across the life cycle. The Excretory/Secretory system represents one mechanism for EV release, which are uniformly internalized by murine macrophages via phagocytosis. The protein components of the vesicles vary in a stage- and sex-specific manner and contain known and putative host immunomodulatory molecules but the asymmetric distribution of these molecules between male and female worms suggests *Brugia*’s ability to modulate the host immune response may have a sexual dimorphic element. Finally, we also show that the process of EV release is inhibited by ivermectin, which not only highlights a mechanism of action for this drug but also suggests vesicle release may be leveraged as an assay platform in future drug discovery efforts.

## Materials and methods

### Profiling extracellular vesicle (EV) release from untreated and ivermectin-treated parasites

All *Brugia malayi* and *Dirofilaria immitis* worms were obtained from the NIAID/NIH Filariasis Research Reagent Resource Center (FR3) at the University of Georgia, USA where *B*. *malayi* infections are maintained in domestic short-haired cats, while *D*. *immitis* infections are maintained in beagle dogs. L3 stage *B*. *malayi* were obtained by FR3 via dissection of cold anesthetized *Aedes aegypti* (black-eye Liverpool strain) 14 days post-infection. More mature stages of *Brugia* spp. were obtained by infecting gerbils (*Meriones unguiculatus*) via intraperitoneal injection of L3, which were then recovered by necropsy. L4 were obtained 10 days post-infection and adults after 3 months post-infection. Collected parasites were cultured at Iowa State University in 50 mL RPMI (Sigma Aldrich, St. Louis MO) at 37°C (5% CO_2_) with spent media replaced every 24 h. EVs were isolated from spent culture media using differential centrifugation as previously described (32) and quantified by Nanoparticle Tracking Analysis (NanoSight LM10, Malvern Instruments, Malvern, UK). EV samples were stored at -80°C.

To examine the effects of ivermectin on EV release, one million *B*. *malayi* mf, 100 L3, 10 adult male and 10 adult female parasites per treatment were cultured as described above for 24 hrs in 1.3 ml, 1.3 ml, 3 ml and 10 ml of RPMI media, respectively, containing either ivermectin (Sigma Aldrich) at 1 μM final concentration or 0.01% DMSO (vehicle control). *Dirofilaria immitis* Missouri and JYD-34 strain L3s were treated in an identical manner. EVs were purified and quantified as described above; one-way ANOVA (Prism 7, GraphPad Software, La Jolla CA) was used to determine significance of drug-induced changes in vesicle release.

### Visualization of EV morphology by electron microscopy

An aliquot of purified EV preparation was mixed with uranyl acetate (2% w/v final concentration), incubated for 5 min at room temperature and applied to carbon-coated copper grids. Images were taken at 80kV using a JEOL 2100 200-kV scanning and transmission electron microscope (STEM) with a Thermo Fisher Noran System 6 analysis system.

### Localization of EV release by immunocytochemistry

Nematodes were fixed in 4% PFA (Electron Microscopy Sciences, Hatfield, PA) in PBS and freeze-cracked by immersing the tube in liquid N_2_ for 2–3 min, thawing in a 37°C water bath and repeating the process twice. They were fixed again in 4% PFA in PBS for 4 hours at 4°C. Worms were washed with PBST (0.1% Triton-X 100 [Sigma-Aldrich] in PBS) and permeabilized overnight in 2-mercaptoethanol solution (5% 2-mercaptoethanol, 1% Triton-X 100, 120 mM Tris, pH 7.0) at 37°C. Following thorough washes in PBST, the worms were incubated in antibody diluent (AbD; 0.1% BSA, 0.1% NaN_3_ [Sigma-Aldrich] in PBST) overnight. Next, they were incubated with anti-Alix (ALG-2-interacting protein X) Rabbit primary antibody (diluted 1:1000 in AbD)(EMD Millipore, Billerica, MA; Catalogue Number ABC40) at 4°C for 2 days, washed in AbD three times and incubated in AbD overnight. The same procedure was followed for the incubation in Alexa Fluor 488 Donkey anti-Rabbit secondary antibody (Invitrogen, Carlsbad, CA; Product Number R37118) at 1:1000 dilution in AbD. Following an overnight wash in AbD, the worms were incubated in 1:100 Alexa Fluor 647 Phalloidin (Invitrogen) and 1:100 Hoechst 33342 (Invitrogen) at 4°C overnight. The worms were washed in AbD the next day and mounted using Flouromount aqueous mounting medium (Sigma-Aldrich) and visualized by a Leica SP5 X MP confocal/multiphoton microscope system. To rule out non-specific binding of secondary antibody, a negative control where primary anti-Alix was omitted was also performed.

### EV uptake by murine macrophages

5 x 10^5^ macrophages (J774A.1) were grown for 24 h on coverslips commercially coated with Poly-D-lysine (Thermo Fisher Scientific). EV preparations from different *B*. *malayi* life stages were stained with PKH67 (Sigma-Aldrich), a green lipophilic dye, according to manufacturer’s instructions. 1 x 10^7^ stained EVs were added to adhered macrophages incubated at 37°C for an additional 24 h. EV uptake was visualized using immunocytochemistry. Media was removed and cells washed with 1X PBS and fixed in 4% PFA. Following three room temperature washes with 1X PBS, cells were incubated in 1:200 Alexa Fluor 647 Phalloidin for 30 min followed by three 10 minute washes with 1X PBS. 1:500 Hoechst 33342 was added to the cells and incubated for a further 10 minutes before being washed twice in 1X PBS. Coverslips were mounted on slides using Flouromount aqueous mounting media (Sigma-Aldrich) and visualized by a Leica SP5 X MP confocal/multiphoton microscope system (Leica Microsystems Inc., Buffalo Grove, IL). Acquired images were processed using Leica Application Suite X software and assembled into 3-D format using Imaris 7.7.1 (Bitplane, Concord, MA), a software program used for rendering three-dimensional surfaces from microscopy image data.

Uptake of fluorescently labeled vesicles was quantified using flow cytometry. Macrophages were grown on coverslips and co-incubated with EV for 24 h as described above. Following co-incubation, media was removed and 1 ml of FACS buffer (0.1% BSA, 0.1% NaN_3_ in 1X PBS) was added. The cells were scraped, collected in 1.5 ml tubes and centrifuged at 250 x g for 10 min at 4°C. The supernatant was removed and cells fixed in 1% paraformaldehyde in FACS buffer before transfer to 5 ml polystyrene tubes for analysis using FACSCanto (BD Biosciences, San Jose, CA). One-way ANOVA (Prism 7) was used to determine the significance of any differences in uptake of different life stage EVs.

EV internalization mechanisms were explored using endocytic inhibitors to selectively block relevant pathways. Cells were incubated in the presence of 200 μM Dynasore, 30 μM Chlorpromazine or 300 μM Genistein (all Sigma-Aldrich) 1–2 hours prior to the addition of PKH67-labeled EVs. Positive controls for these inhibition experiments included co-incubation of select inhibitors with either 0.1 μm Fluoresbrite Carboxylate Microspheres (PolySciences Inc., Warrington, PA), Alexa Fluor 555 conjugated transferrin (Life Technologies) or Alexa Fluor 555 conjugated Cholera toxin subunit B (Life Technologies). Again, immunocytochemisry 24 h post-treatment was used to image EV internalization and flow cytometry used to quantify uptake.

### Proteomic analysis of EV proteins

Proteomic analysis of EV preparations was performed commercially (System Biosciences, Palo Alto, CA). Briefly, purified EV preparations were resuspended in RIPA Buffer (2.0% SDS, 150 mM NaCl, 50 mM Tris, pH 8.5, 1X Roche cOmplete Protease Inhibitor Cocktail [Sigma-Aldrich]) and heated at 100°C for 15 minutes, clarified by centrifugation and the protein concentration determined by Qubit fluorometry (Invitrogen). 8–10 μg of protein was processed by SDS-PAGE using 10% Bis-Tris NuPage mini-gel (Invitrogen). Following in-gel tryptic digestion at 37°C for 4 h, samples were analyzed by nano LC-MS/MS with a Waters NanoAcquity HPLC System (Waters Corp. Milford, MA) interfaced to a ThermoFisher Q Exactive. Spectral data were searched against copies of the UniProt *B*. *malayi*, *Ascaris suum* and *Caenorhabditis elegans* databases (UniProt release 2017_4) using a locally running copy of MASCOT v2.6 (Matrix Science Ltd., London, UK). Common mammalian contaminants were also included in this step and positive matches removed from downstream analysis. The search was restricted using the following parameters; maximum missed cleavages = 2, fixed modifications = carbamidomethyl (C), variable modifications = Oxidation (M), Acetyl (N-term), Pyro-Glu (N-term Q) and Deamidation (N, Q), a peptide mass tolerance of 10 ppm, and a fragment mass tolerance of 0.02 Da. Mascot DAT files were parsed into the Scaffold software for validation, and filtered to create a non-redundant list from which mammalian contaminants were subtracted. Data were filtered using 1% protein and peptide FDR and requiring at least two unique peptides per protein. Protein sets were compared using UpSetR (40), a novel web-based technique to analyze discrete data sets and their intersections. GO analysis was performed using Blast2GO v.4 (BioBam Bioinformatics, Valencia, Spain), a program that extracts GO terms from remote BLAST searches (https://blast.ncbi.nlm.nih.gov/Blast.cgi) of input queries and from InterPro motifs identified via InterProScan (www.ebi.ac.uk) to generated a list of validated GO annotations. The transmembrane topology of identified proteins was predicted using locally installed HMMTOP v.2.1.

## Results

### Extracellular vesicles (EVs) are released throughout the *Brugia* life cycle

**Fig 1:**
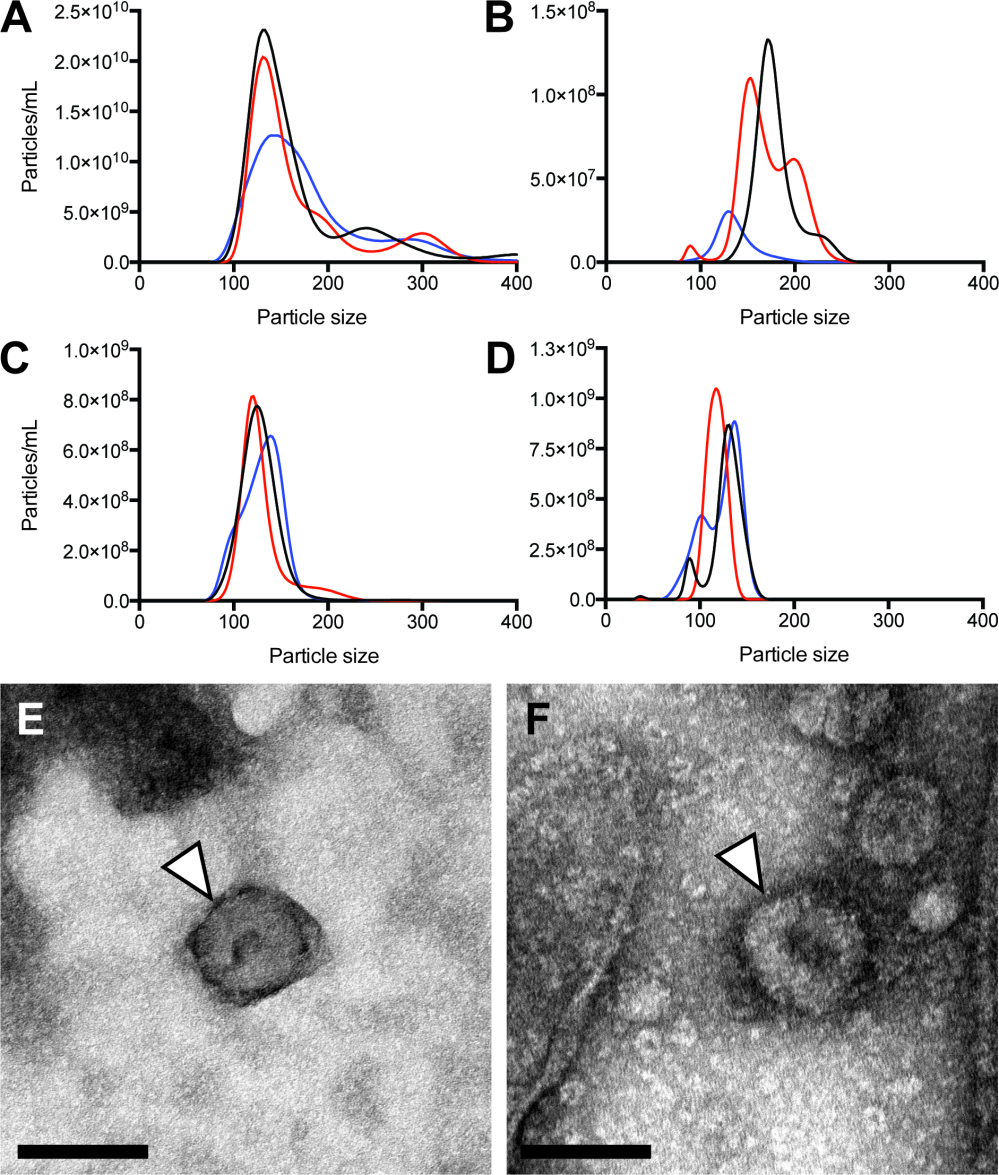
All *B*. *malayi* intra-mammalian life cycle stages release extracellular vesicles (EV). (A-D) EVs were isolated from spent culture media then sized and quantified using nanoparticle tracking analysis (NTA). NTA of EV preparations from three individual 24 hr cultures of microfilariae (A), L4 (B), adult male (C) and adult female parasites (D) are shown. Particle size is in nm. (E-F) Electron micrograph of representative adult male and female EV (white arrowheads), scale bar 100 nm.

We previously described the highly abundant release of a discrete EV population from infective stage (L3) *B*. *malayi* [32]. EV release from adult male and female *Brugia*, in contrast, was not definitive and led to the hypothesis that EV secretion is primarily a process utilized by *B*. *malayi* larvae. To test this hypothesis we examined EV release from all other intra-mammalian life stages of *B*. *malayi*. Microfilariae (mf), L4, adult male and adult female worms were incubated in culture media from which any secreted EVs were purified and quantified using previously published protocols [32]. All life stages examined were found to release extracellular vesicles of a size consistent with exosomes (Fig 1A–1D). The size distribution of the vesicle population ranged from 85–200 nm diameter, which is consistent with that reported previously for L3 stage parasites [32] and vesicles released by other parasitic nematode species [31,33,41]. Most variation in vesicle size and output was noted for L4 stage worms (Fig 1D) although in general, there was little variation in vesicle secretion profile between individual parasites. Morphology of the parasite-derived vesicles was examined using negative stain electron microscopy. Again, consistent with our observations of *B*. *malayi* L3 EVs, vesicles secreted by these other intra-mammalian life stages are round single vesicles [42] that exhibit the accepted “cup-shaped” or “donut” morphology of exosome-like vesicles visualized via this method [43] (Fig 1E and 1F).

### The Excretory-Secretory pore of microfilariae is a candidate site for EV release

**Fig 2:**
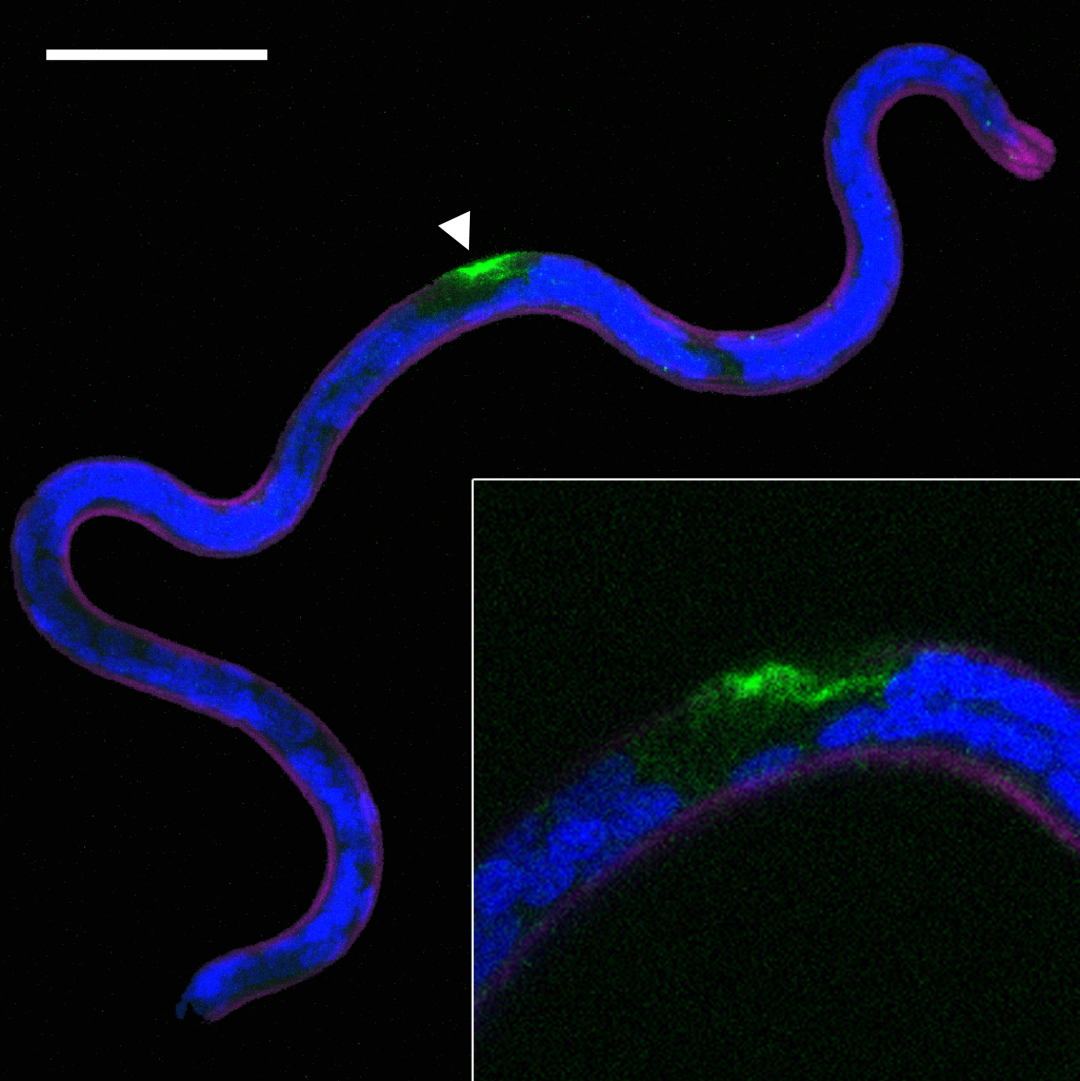
*B*. *malayi* microfilariae release EV from the excretory/secretory (ES) pore. Confocal scanning laser micrograph showing anti-Alix immunoreactivity (IR, green) focused at the ES pore (white arrowhead). Alix is frequently found in EV proteomes and considered an EV marker. Anti-Alix IR can be observed extending from the ES pore within a duct-like structure (inset). Worms were counterstained with Hoechst 33342 (nuclei, blue) and phalloidin (muscle, purple). Scale bar 20 μm.

The mechanism by which exosomes are released by parasitic nematodes is unclear. To identify a site for EV secretion by *B*. *malayi* we employed whole-mount indirect immunocytochemistry (ICC) interfaced with confocal scanning laser microscopy using primary anti-Alix antibodies. ALG-2-interacting protein X (Alix) is an ESCRT associated protein found in many mammalian exosomes and is widely accepted as a mammalian exosomal marker [44–47] although it has not been validated as an EV marker in *Brugia* or parasitic nematodes more generally. An Alix homolog is present in the *Brugia* genome (UniProtKD ID A0A0K0JIH7) that lacks a signal protein such that identification of extracellular anti-Alix immunoreactivity would suggest a non-traditional pathway for Alix secretion, such as EV. Low-level punctate anti-Alix immunoreactivity was observed in *B*. *malayi* microfilariae preparations with intense immunoreactivity focused around the Excretory-Secretory (ES) pore (Fig 2) and its associated duct (Fig 2, inset) suggesting this structure might be the site of EV release in microfilariae. The ES pore is the terminus of the parasite excretory system that may be considered functionally equivalent to the vertebrate renal system. No focused anti-Alix immunoreactivity was observed in either L3 or adult preparations. It may be that EVs released by these stages do not express Alix, or that a technical obstacle in whole mount ICC in the larger worm stages proved obstructive to vesicle localization. Subsequent profiling of adult male and female EV proteomes (see Table 1, S1 and S2 Tables) did not identify Alix in those vesicles, and Alix was not found in a previous profile of L3 EV proteins [32], suggesting alternative target proteins may be more informative EV markers in these life stages; further studies will be required to unequivocally determine routes of EV secretion across the life cycle.

**Table 1:** Most abundant proteins in *B*. *malayi* adult EV proteome.

	Adult male			Adult female	
	Identified Proteins	UniProtKB ID		Identified Proteins	UniProtKB ID
1	Actin (BMS-ACT-5)*	A0A0K0JJB8_BRUMA	1	Galectin (BMA-LEC-2)	A0A0H5S1P8_BRUMA
2	Bm4628, putative ubiquitin	A0A0K0JFG9_BRUMA	2	Actin (BMS-ACT-5)*	A0A0K0JJB8_BRUMA
3	Rab 11 (BMA-RAB-11.1) small GTPase	A0A0K0JE46_BRUMA	3	Annexin	A0A0K0J0N3_BRUMA
4	Rap-1 (BMA-RAP-1) small GTPase	A0A0K0J5Y2_BRUMA	4	Galectin (BMA-LEC-1)	A0A0K0JMF7_BRUMA
5	HSP70 (BMA-HSP-1) heat shock protein	A0A0I9N5E9_BRUMA	5	Triosephosphate isomerase (BMA-TPI-1)	A0A0J9YA50_BRUMA
6	Rab 1 (BMA-RAB-1) small GTPase*	A0A0H5S8N4_BRUMA	6	Macrophage migration inhibitory factor homolog	MIFH_BRUMA
7	Tubulin beta chain	A0A0K0IVQ1_BRUMA	7	ERM family protein (BMA-ERM-1)	A0A0K0J3J0_BRUMA
8	Arf-1 (BMA-ARF-1.2) small GTPase	A0A0J9XQK0_BRUMA	8	Ubiquitin (BMA-UBQ-2)	A0A0H5SEE1_BRUMA
9	Bm13659, putative Ras-1 small GTPase	A0A0H5BRY3_BRUMA	9	HSP70 (BMA-HSP-1) heat shock protein	A0A0I9N5E9_BRUMA
10	Tubulin alpha chain	A0A0K0JYJ6_BRUMA	10	Rab 8 (BMA-RAB-8) small GTPase	A0A0I9R394_BRUMA
11	Gpa-16 (BMA-GPA-16) G-protein alpha subunit	A0A0H5SLI6_BRUMA	11	Bm4259, putative 14-3-3 family protein	A0A1P6C0L5_BRUMA
12	Bm9583, putative 14-3-3 family protein	A0A0K0JZS7_BRUMA	12	Rab 1 (BMA-RAB-1) small GTPase*	A0A0H5S8N4_BRUMA
13	Bm5196, putative elongation factor 1-alpha	A0A0J9Y962_BRUMA	13	Uncharacterized protein	A0A0K0IXP0_BRUMA
14	Bm3367, uncharacterized membrane-associated protein	A0A158PZ00_BRUMA	14	OV25-1 small heat shock protein	A0A0K0JDW5_BRUMA
15	Gpb-1 (BMA-GPB-1) G-protein beta subunit	A0A0K0J9V3_BRUMA	15	Thioredoxin peroxidase 2	A0A0K0J9D6_BRUMA

Proteins extracted from adult male and female EVs were subject to nano-scale LC-MS/MS. Spectral data were searched against the *B*. *malayi*, *Ascaris suum* and *Caenorhabditis elegans* UniProt databases using MASCOT to identify proteins. The 15 most abundant proteins in each preparation (ranked by normalized spectral abundance factor) are shown with corresponding UniProtKB identifiers.

*indicates proteins are in top 15 most abundant proteins of both the male and female EV preparations.

### Murine macrophages internalize *Brugia* EVs by phagocytosis

**Fig 3:**
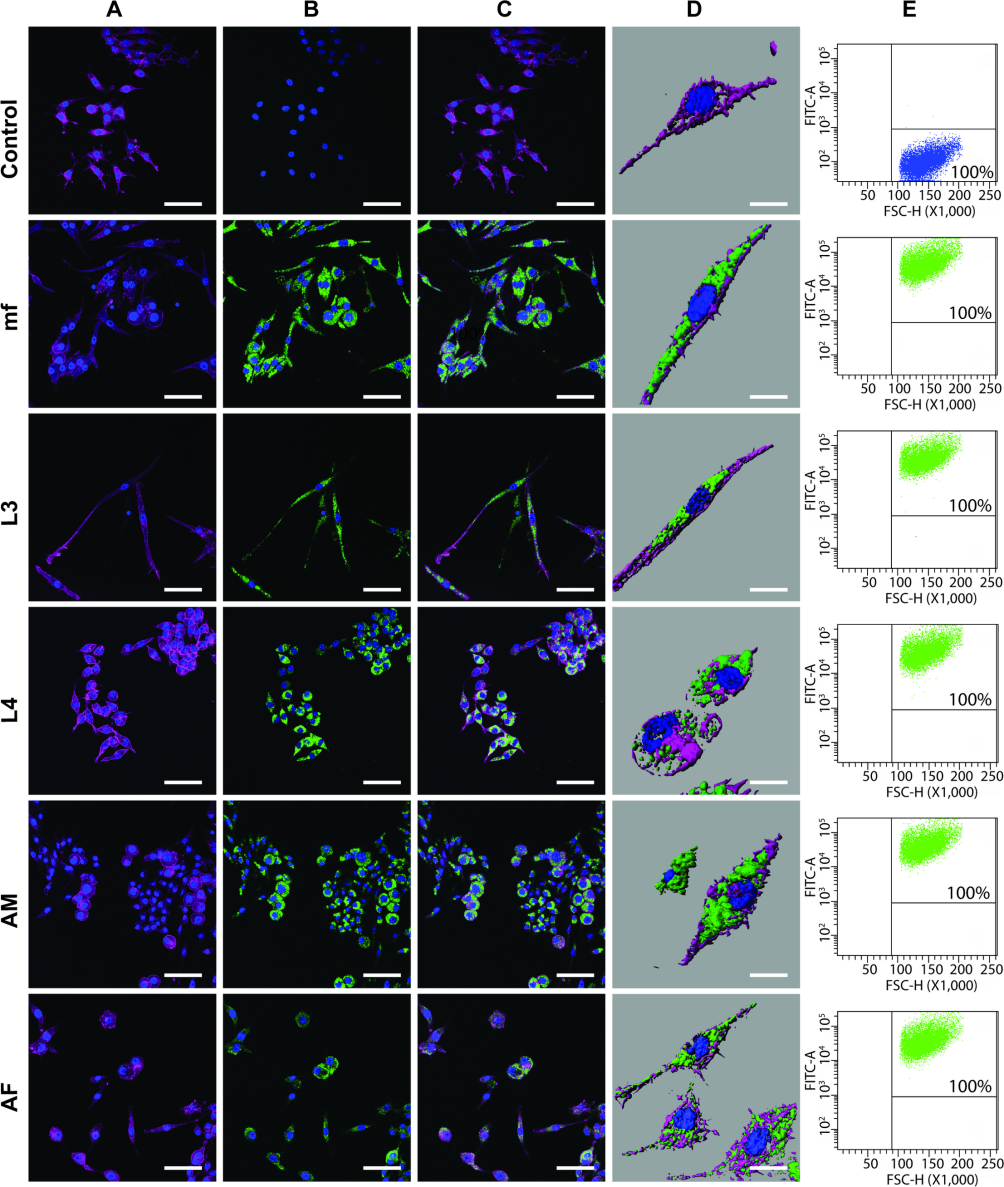
*B*. *malayi* EV are internalized by murine macrophages. (A-C) Confocal scanning laser micrographs showing: (A) murine J774A.1 macrophages stained with Hoechst 33342 (nuclei, blue) and phalloidin (muscle, purple); (B) macrophages following incubation with a no EV control or 1 x 10^7^ PKH67-stained EVs (green) isolated from microfilaria (mf), L3, L4, adult male (AM) and adult female (AF) worms; and (C) an overlay of panels A and B showing EV internalization. All images captured at magnification 63X, all scale bars 10 μm. (D) Imaris 3D reconstruction of confocal micrographs showing labeled EV are internalized and not simply adhered to cell surface. All Imaris images captured at magnification 68X, all scale bars 2 μm. (E) Flow cytometry showing all treated macrophages internalized labeled parasite EVs.

Previously we reported that EVs released by *B*. *malayi* L3 stage parasites are internalized by macrophages [32]. Here we wanted to test whether all life stage EVs (mf, L3, L4, adult male and adult female) are internalized by macrophages and identify a mechanistic basis for this internalization. The interaction of fluorescently labeled *B*. *malayi* EVs with J774A.1 murine macrophages was visualized using confocal microscopy and quantified with flow cytometry. EVs released by all parasite life stages are taken up by murine J774A.1 macrophages (Fig 3). Use of confocal microscopy and Imaris 3D reconstruction software (Bitplane, Concord MA) confirms internalization of labeled EVs and not simply surface adherence of the vesicles to macrophages (Fig 3D). This pattern of internalization was universal, flow cytometry revealed all macrophages treated with EVs took up the parasite vesicles (Fig 3E) and there was no quantitative difference in relative uptake of the different life stage EVs after 24 h co-incubation (S1 Fig). These data suggest that mechanisms of parasite vesicle uptake in the time frame examined are either independent of factors expressed on the EV surface or, if a vesicle derived factor does determine the interaction, that this factor must be conserved across all parasite stage vesicles. Further, the uniform uptake of all parasite stage vesicles described in this experiment would indicate that any downstream variation in host cell response to different *Brugia* life stage EVs is more likely to result from the properties of vesicle cargo or membrane-bound effectors than simply differential internalization of the vesicles themselves.

**Fig 4:**
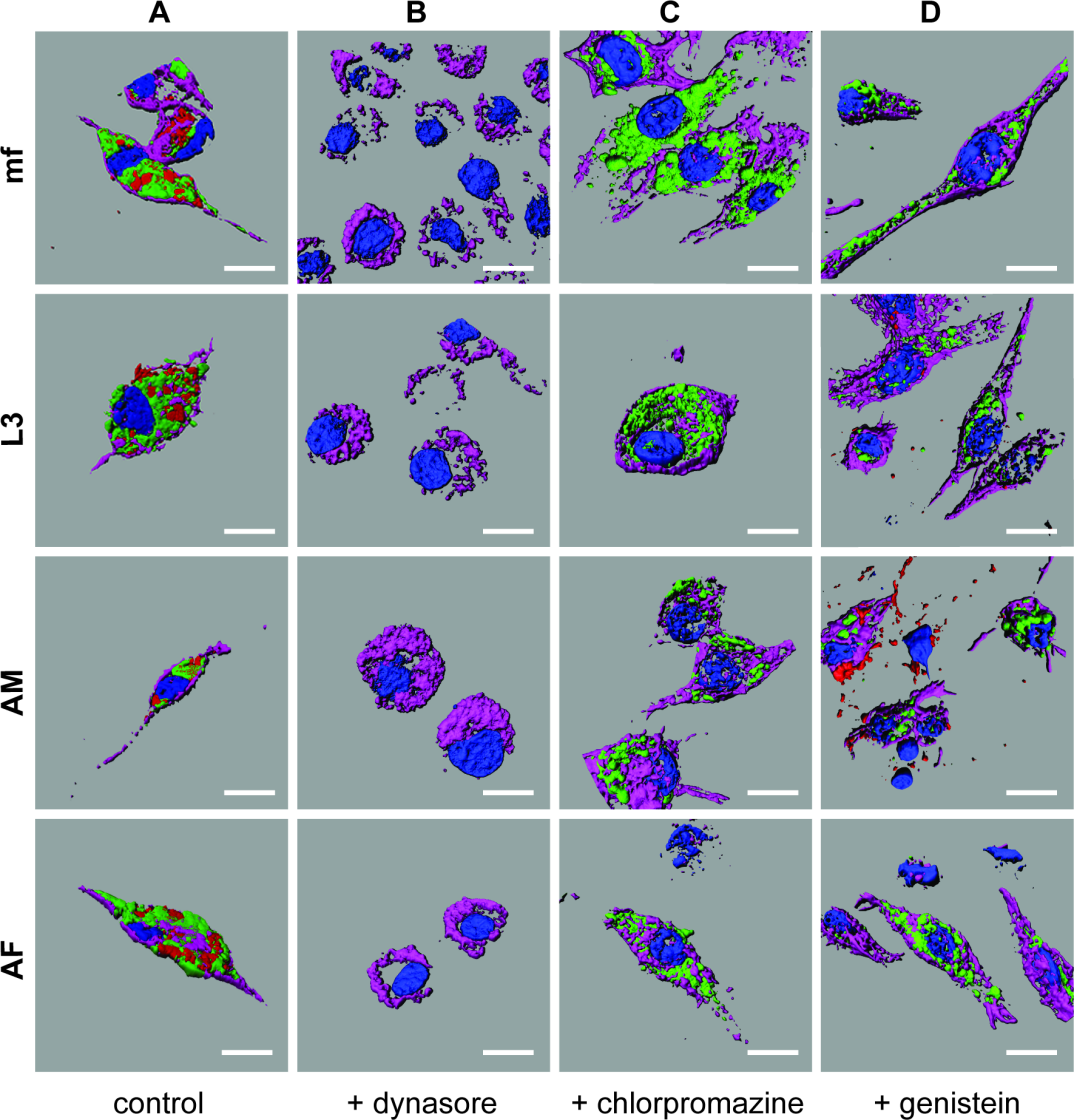
Murine macrophages internalize parasite-derived EV by phagocytosis. Imaris 3D reconstructed confocal micrographs of murine J774A.1 macrophages. (A) Control macrophages showing internalization of PKH67-labeled EV (green) isolated from microfilaria (mf), L3, adult male (AM) and adult female (AF) worms in parallel with Fluoresbrite Carboxylate Microspheres (red, phagocytosis tracer). Macrophages are counterstained with Hoechst 33342 (nuclei, blue) and phalloidin (muscle, purple). (B) Macrophages treated with labeled EV (green) and microspheres (red) in the presence of 200 μM Dynasore. Absence of green and red indicates internalization of both EV and tracer are blocked. (C) Macrophages treated with labeled EV (green) and Alexa Fluor 555 conjugated transferrin (tracer, red) in the presence of 30 μM Chlorpromazine. Presence of green and absence of red indicates internalization of tracer is blocked but EV is not. (D) Macrophages treated with labeled EV (green) and Alexa Fluor 555 conjugated cholera toxin b (tracer, red) in the presence of 300 μM Genistein. Presence of green and general absence of red indicates internalization of tracer is generally blocked but EV is not. All Imaris images captured at magnification 68X, all scale bars 2 μm.

In an effort to characterize the mechanisms of this EV internalization, J774A.1 macrophages were pretreated with chemical inhibitors of discrete internalization pathways before incubation with fluorescently labeled parasite EVs. This approach of selective vesicle uptake inhibition suggests *Brugia* EVs are internalized by phagocytosis (Fig 4). Dynasore is a fast-acting small molecule inhibitor of dynamin GTPase and therefore an inhibitor of dynamin-dependent endocytic pathways [48]. Pretreatment with dynasore completely blocked internalization of both fluorescent carboxylate microspheres (a positive control for phagocytosis) and all *B*. *malayi* life stage vesicles examined (Fig 4B). This strongly suggests that EV uptake occurs via dynamin-dependent processes, that is to say, via either phagocytosis, clathrin-mediated endocytosis or caveoli-mediated endocytosis (or a combination thereof). Pretreatment of macrophages with chlorpromazine, which selectively blocks clathrin-mediated endocytosis [49], inhibited internalization of fluorescently-labeled transferrin (a ligand of clathrin-mediated endocytosis) but did not block the uptake of any stage-specific parasite vesicles (Fig 4C). Lastly, genistein, an inhibitor of caveolin-dependent internalization [50], did not block parasite-derived vesicle uptake but did effectively inhibit fluorescently labeled cholera toxin (CTX), a marker of caveolae- and raft-mediated pathways (Fig 4D).

### Vesicle proteomic profiling identifies proteins involved in exosome biogenesis and putative immunomodulators

In an attempt to identify indicators of vesicle function, we examined the protein content of *Brugia* EVs. 26.66 μg protein was extracted from adult male EV and 18.95 μg total protein from adult female EV for analysis. A total of 20 distinct *B*. *malayi* proteins were identified in the adult male EV preparation (S1 Table) and 74 in the adult female preparation (S2 Table). A summary of the 15 most abundant proteins in each sample set is presented in Table 1. The combined *B*. *malayi* adult EV proteome contains proteins that are involved in exosome biogenesis suggesting that the secreted parasite EV profile (Fig 1) contains, at least in part, an exosome-like component. Examples of such molecular markers include small GTPases such as Rab 11 and Rab 27, critical molecules in the exosome biogenetic pathway [51,52], ARF1 and annexins [53]. Whilst generally supporting a designation of these vesicles as “exosome-like”, the identification of canonical exosome markers is not equivocal and is discussed later. To probe the nature of protein association with EVs, all proteins were subjected to transmembrane topology prediction using HMMTOP. 29 of the 74 (39%) adult female EV proteins were predicted to contain at least one transmembrane domain compared to 8 of 20 (40%) adult male EV proteins. These data suggest approximately 40% of adult EV proteins may associate with the vesicle membrane and therefore may derive from the surface membrane of the parent cell. It follows that 60% of EV proteins are putatively cytosolic and may include proteins actively loaded into the vesicles. The same analysis on the whole predicted *Brugia* proteome yields similar ratios (5,074 of 13,435 predicted proteins, or 38%, contain at least one putative transmembrane domain) indicating that there is no overall enrichment of either membrane-associated or cytosolic proteins in secreted EV.

**Fig 5:**
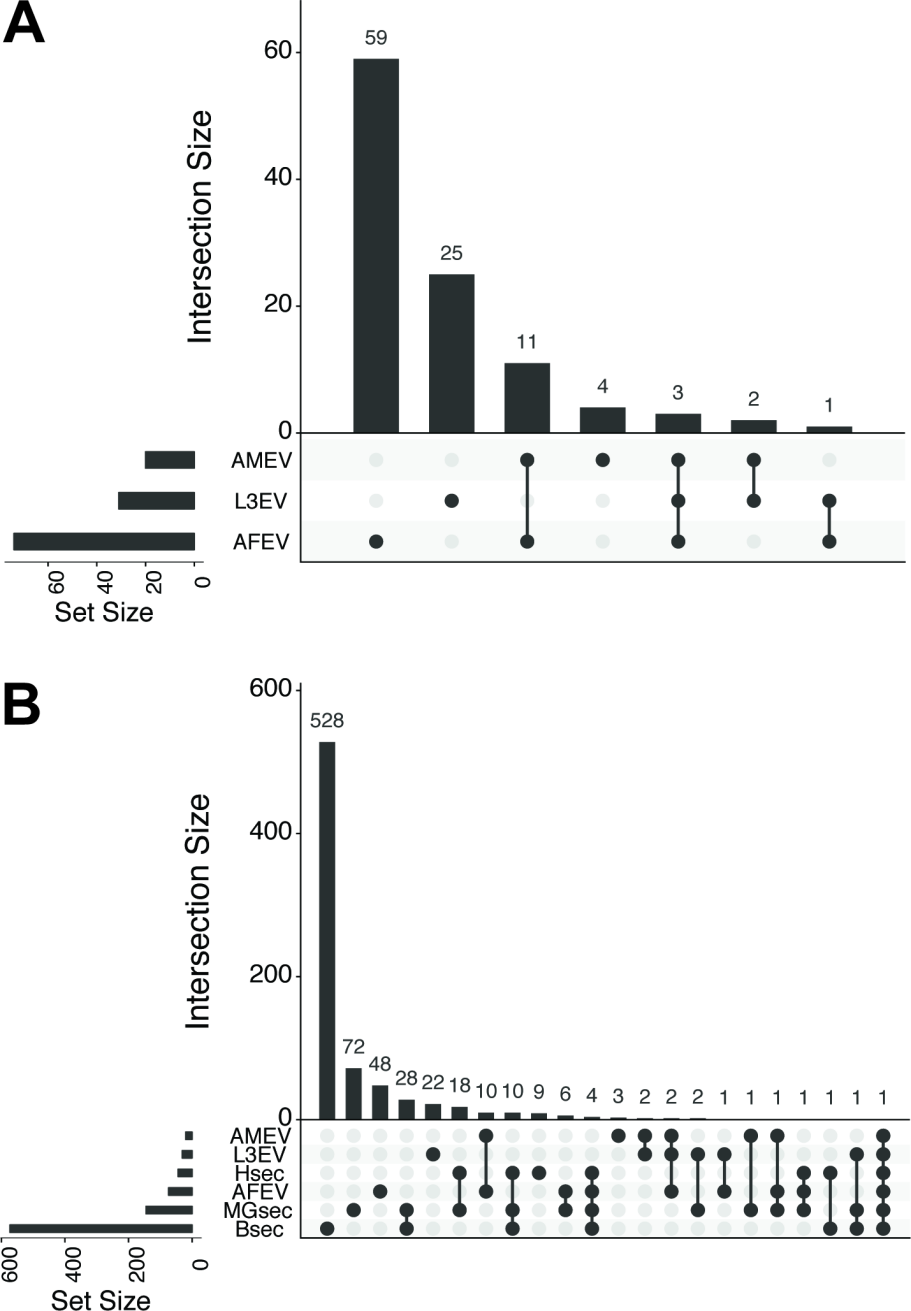
*Brugia* EV proteome is largely stage- and sex-specific. (A) UpSetR analysis visualizing intersections between adult male (BmAM), female (BmAF) and previous L3 (BmL3) EV proteome datasets. Datasets participating in intersections are shown as filled (black) circles, the number of unique proteins in that intersection is presented in the histogram above. For example, 59 EV proteins are unique to BmAF but 11 are shared between BmAF and BmAM only. (B) Extended UpSetR analysis to include previously published *B*. *malayi* secretomes. Secretome appellation reflects lead authors Hewitson (Hsec)(12), Moreno and Geary (MGsec)(14), and Bennuru (Bsec)(14).

Protein profiles of adult male and female EV, along with the L3 stage EV profile previously described [32], were compared using UpSetR [40] to identify trends and intersections between the three data sets (Fig 5A). The protein profile of adult female and L3 vesicles was found to be generally specific to that stage or sex. 59 of the 74 proteins identified in the adult female preparation (80%) are only found in that set, whilst 25 of the 31 L3 EV proteins (81%) were specific to that profile. There was much greater intersection in the adult male sample, with only 4 of the 20 (20%) proteins identified specific to that preparation. The two sets with the highest similarity were the two adult preparations; 11 vesicle proteins were uniquely conserved between the male and female EV profiles. Only three proteins were found in all three of the *Brugia* EV profiles: Rab 1, HSP70 and actin. Expansion of the UpSetR analysis to include published *B*. *malayi* secretomes [12–14] reveals further trends (Fig 5B). Despite the addition of these data sets, the pool of unique proteins in the adult female EV profile and L3 profile remained largely unchanged (48 proteins identified only in the adult female EV and 22 in the L3 EV). Thus, proteins in *Brugia* EV proteome profiles typically do not appear in the whole secretome and are not a subset of it. It is likely that differences in sample preparation account for this observation; EV samples are lysed in RIPA buffer prior to tryptic digestion whereas secretome samples are not [12–14]. Without this lysis step, EV proteins may be protected against tryptic digestion within the vesicle and would not be incorporated for downstream processing. [N.B. one caveat to such cross–omic analysis is that not all putative secreted proteins correlated with previous studies. Some proteins identified via gene name IDs from EST datasets or now deleted Uniprot and TIGR Accession numbers do not map to the new UniprotKB predicted *Brugia* proteome based on the most recent draft genome. For example, only 422 out of 775 gene name IDs from the consolidated whole *Brugia* secretome [14] successfully mapped to 574 UniprotKB IDs. Irrespective, this partial dataset provides a valuable comparison between freely secreted and vesicle-packaged proteins.]

**Fig 6:**
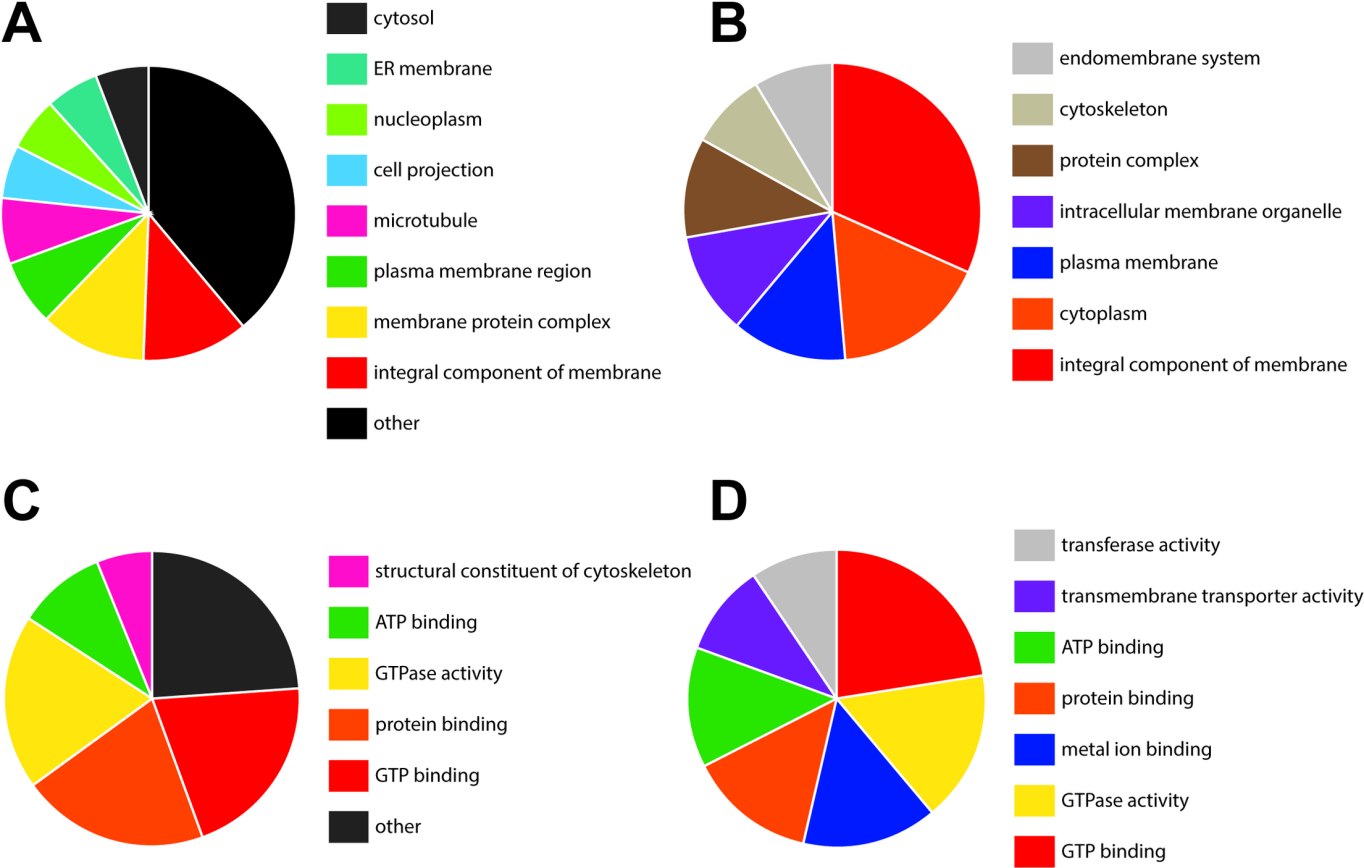
*Brugia* EV proteome contains markers of exosome biogenesis. (A-B) Gene Ontology (GO) analysis of adult male (A) and female (B) EV proteomes summarizing cellular location GO terms, including terms associated with the endosomal pathway. (C-D) GO analysis of adult male (C) and female (D) EV proteomes summarizing molecular function GO terms.

GO analysis was used to categorize and compare protein function and cargo of *B*. *malayi* adult male and female EVs. A summary comparison of male and female vesicle protein GO annotations binned according to cellular component and molecular function are shown in Fig 6; full hierarchical GO graphs are presented in S2–S5 Figs. All 20 adult male EV proteins and 71 of the 74 adult female EV proteins were annotated with at least one GO term. In both male and female EV profiles, GO terms associated with the plasma membrane are common, reflecting the membrane origin of all EV whether they be true exosomes or microvesicles [43,54]. Regarding the specific identity of these vesicles, GO terms associated with the endosome (GO:0005768) and the endomembrane system (GO:0012505) were represented in both the male and female samples, pointing to an endosomal biogenesis for at least a cohort of the secreted vesicles. GO terms associated with cellular component (Fig 6A and 6B) were more divergent between male and female preparations but still reflected the vesicular nature of the samples. Examples include GO:0031410 (cytoplasmic vesicle), GO:0097708 (intracellular vesicle) and GO:0070062 (extracellular exosome) in the male preparation, and GO:0044446 (intracellular organelle) and GO:0043231 (intracellular membrane bound organelle) in the female preparation. GO analysis was also used to glean clues to the function of these vesicles and a higher degree of similarity between the male and female preparations was noted when GO terms were binned according to Molecular Function (Fig 6C and 6D). Both male and female preparations shared a high proportion of GO terms associated with hydrolase activity including GTPase (GO:0003924) and ATPase (GO:0016887), and ribonucleoside binding, both GTP binding (GO:0005525) and ATP binding (GO:0005524). These terms align closely with the number of small G-proteins involved in exosome biogenesis identified in the EV proteomes and similar terms have been described as enriched in exosome samples elsewhere [44].

Our overall hypothesis is that EV released by filarial nematodes modulate host biology to facilitate parasite infection. We examined the *Brugia* EV proteome in a more itemized manner for specific proteins that have documented immunomodulatory activity or that are known to be involved in parasite pathogenesis or virulence. Thirteen proteins (18%) in the adult female EV proteome met these criteria, for example, the parasite cytokine macrophage migration inhibitory factor homolog (MIF-1/BmMIF-1), glycan-binding proteins (galectins 1 and 2), heat shock proteins, thioredoxin peroxidases and GAPDH. In contrast to the female sample, only three potential effectors were found in the male EV proteome, all in the second category; heat shock proteins -70 and -90, and ATP synthase (whilst not a well-documented immunomodulator, it has been shown that when macrophages engulf EV containing ATP synthase, their bioenergetics are altered [55]). Both the heat shock proteins were also found in the female sample. These results suggest that the female EV proteome is enriched in immunomodulatory or pathogenesis-related effectors.

### Ivermectin inhibits EV release from filarial nematodes

**Fig 7:**
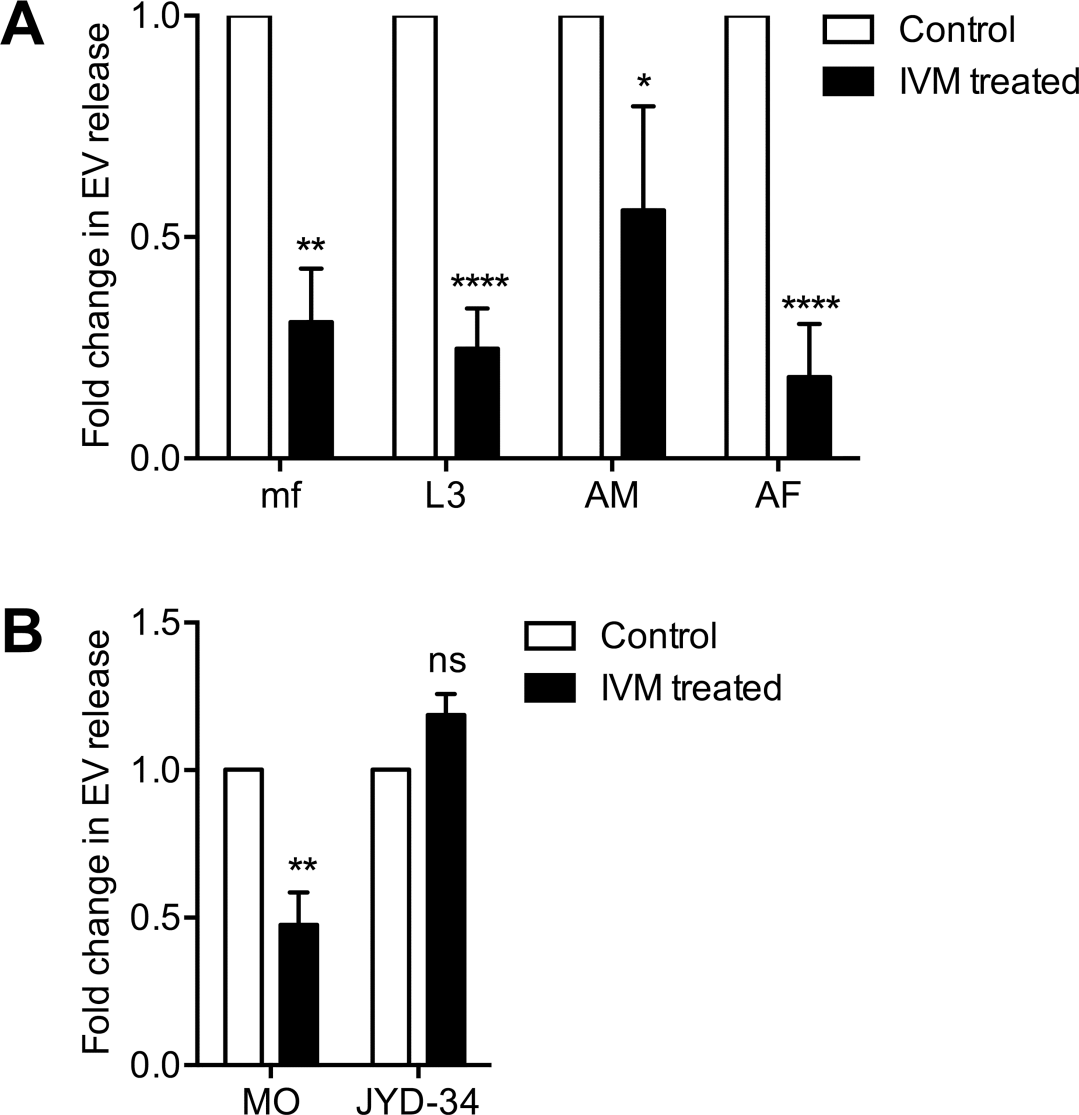
Ivermectin inhibits EV release from filarial nematodes. (A) 1 μM IVM reduces EV release by *B*. *malayi* in vitro. Microfilaria (mf), L3, adult male (AM) and female (AF) were incubated in RPMI containing IVM or vehicle control. Media was collected after 24 hr and EV isolated and quantified. N = 3 (minimum), mean ± SEM, *P<0.05, **P<0.01, ****P<0.0001. (B) 1 μM IVM reduces EV release in vitro by IVM susceptible Missouri strain (MO) *D*. *immitis* L3 but not IVM reduced susceptibility JYD-34 strain. N = 3 (minimum), mean ± SEM, **P<0.01, ns not significant.

Moreno et al. [56] recently observed that ivermectin (IVM) rapidly inhibits protein release from the excretory pore of *B*. *malayi* mf and reported glutamate-gated chloride channel immunoreactivity in close association with musculature around said structure. This work pointed to a new mode of action for ivermectin, the inhibition of protein release from mf stage parasites. Our observation that EVs are also released from the excretory pore of mf stimulated the hypothesis that IVM would similarly inhibit EV release. To test this hypothesis we treated mf, L3, adult male and adult female *B*. *malayi* with a single dose 1 μM concentration of IVM and quantified EV release over a 24 hr culture period. IVM inhibits EV release from all *B*. *malayi* life stages examined (Fig 7A); EV release from mf was inhibited by 70% (P<0.01), L3 by 75% (P<0.0001), adult males by 44% (P<0.05) and females by 82% (P<0.0001). There was no statistically significant difference in IVM inhibition between life stages or sexes but the reduced IVM efficacy against adult males compared to females and other stages and might suggest some sex-specific effects that warrant further investigation. These quantitative reductions in EV release align closely with reported values for IVM inhibition of protein release from mf [56]. To support the idea that this EV phenotype is specific to IVM and not due to toxicity or off-target effects, we recapitulated the experiment using two field isolates of the related filarial nematode, *Dirofilaria immitis*. Missouri (MO) strain is susceptible to therapeutic doses of IVM but the JYD-34 strain has reduced IVM susceptibility [57,58]. 24 hr incubation with 1 μM IVM inhibited EV release by over 50% (p < 0.005) from L3 stages of the IVM susceptible MO strain (Fig 7B), however, IVM had no inhibitory effect on EV release from JYD-34 parasites, suggesting that the inhibitory IVM phenotype is specifically related to the action of the drug rather than a consequence of toxicity. The effect of IVM on *Brugia* and *Dirofilaria* motility was not assayed in parallel to EV release.

## Discussion

**Fig 8:**
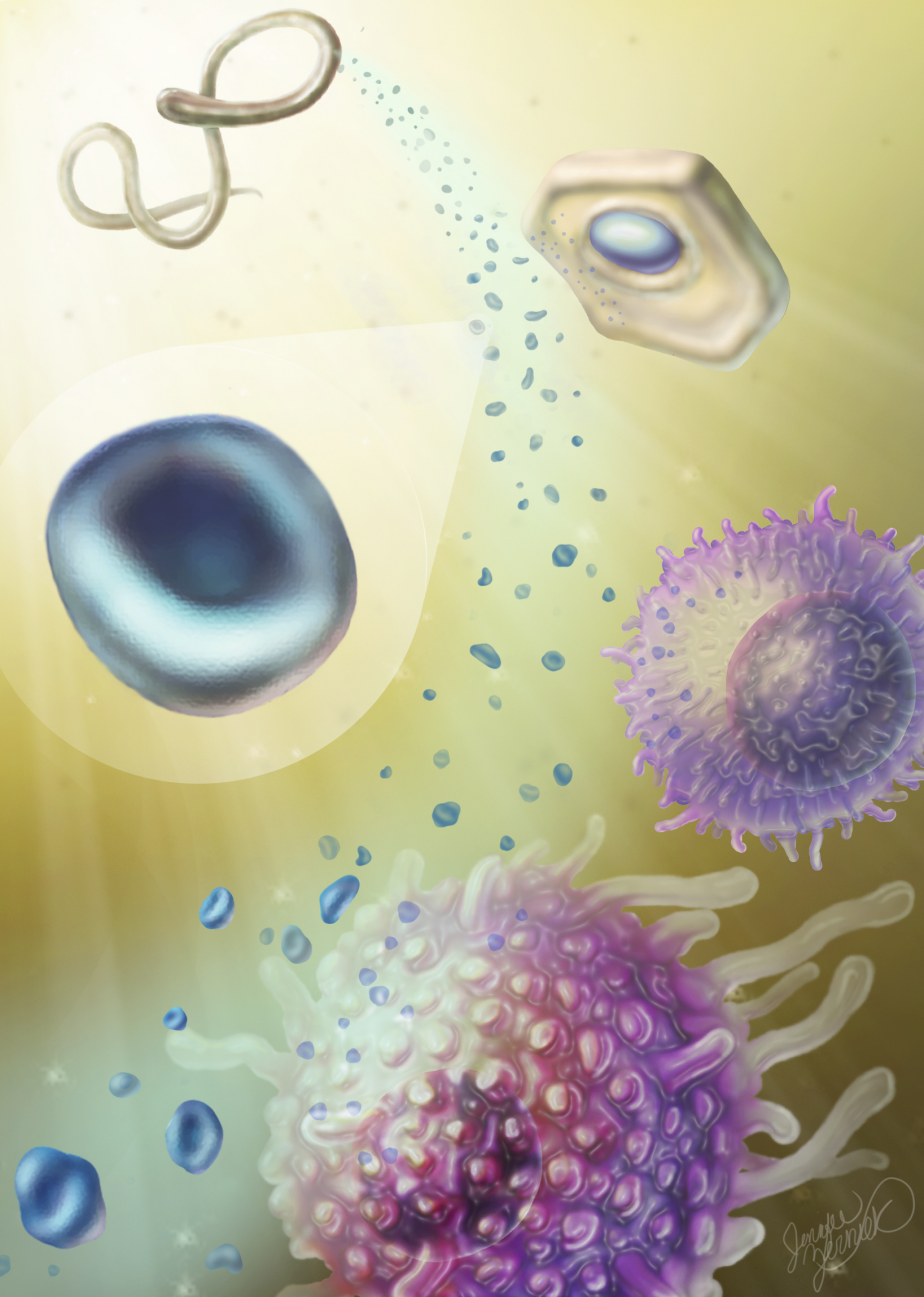
Filarial nematodes release EVs that are relevant at the host parasite interface. Our model proposes that *B*. *malayi* (and *D*. *immitis*) release EV from structures including the excretory pore. These EV (magnified) are of a size and morphology consistent with exosomes but lack some canonical vertebrate exosome markers. They do, however, contain immunomodulatory effector proteins and are phagocytosed by vertebrate cells such as macrophages and potentially other relevant cell types (pictured), providing a mechanism by which these nematodes may deliver effector molecules and manipulate the host.

There is an emerging body of evidence that parasitic worms, both nematodes and platyhelminths, release exosomes into the host milieu and that these exosomes contain potential effector molecules that may function at the host parasite interface. Our overarching hypothesis is that filarial nematodes, including *B*. *malayi*, use EVs to release effector molecules that facilitate the establishment or maintenance of parasitemia (Fig 8). In this study we have examined EV release across the *Brugia* life cycle, describing aspects of vesicle release, interaction with murine macrophages, and structural or cargo components.

We use the catchall term extracellular vesicle (EV) to categorize the vesicles released by all *Brugia* life stages examined. These *Brugia* EV are consistent with exosomes in terms of their size and morphology but without further work to define how and where these EV are generated, calling them exosomes is premature. The proteomic profiling performed here on the adult male and female EV advances our understanding of *Brugia* EV form and function, and provides some insight into the question of what they actually are. The identification of proteins involved in exosome biogenesis suggests at least a cohort of the potentially heterogenous *Brugia* EV population is exosomal-like. The EV proteome was enriched in small GTPases involved at numerous junctures in exosome biogenesis and release. For example, ADP ribosylation factor 6 (ARF6) regulates invagination of late endosomes and the formation of multivesicular bodies (MVB) [59], Rab11 modulates the fusion of MVBs to the plasma membrane of reticulocytes and therefore is critical for extracellular release of exosomes [51,60], and Rab27 (a and b) contributes to exosome release from cells by regulating the interaction between MVB and the cell surface [52]. Broadening this scope, exosomes from widely divergent cell types and organisms are enriched in certain proteins [61] (ExoCarta exosome protein database, www.exocarta.org) constituting a list of proteins with conserved roles in “exosome” maturation and release and which effectively represents a potential compendium of exosome marker molecules. Numerous *Brugia* EV proteins are represented in this enriched protein list (for example Hsp70, elongation factor 1a, actin, annexins etc). However, simply the presence of these proteins is not sufficient support to define the EV population. Refinements in purification methods interfaced with proteomic profiling show that not only are EV populations markedly heterogeneous but that there is notable overlap in both EV subtype size range and enrichment of “exosome markers” across the various EV subtypes [62–69]. Kowal *et al*. [46] found many putative markers are present across multiple, if not all, EV subtypes and that “bona fide” exosomes could only be defined by the presence of CD63, CD9 and CD81. None of these markers were found in the *Brugia* EV proteome and a cursory BLAST analysis reveals the *Brugia* genome does not encode these proteins. Thus *Brugia* does not secrete (and may not be able to generate) true exosomes according to the mammalian characteristics of these vesicles. That does not preclude other small EV subtypes being functionally homologous or relevant and the application of more refined isolation methods in combination with proteomic and functional analyses will shed light on the precise nature of *Brugia* EV.

The ability of *Brugia* to modulate host biology and the role of parasite-derived proteins is well established and reviewed [17,19–21,70]. The presence of immunomodulatory proteins in the EV proteome would indicate this is a mechanism for targeted delivery of parasite effectors to host tissues. To this end, *Brugia malayi* macrophage migration inhibitory factor-1 (BmMIF-1) is well-characterized effector protein [71–73] and likely contributes to the development of the Th2 type environment observed with filarial infection. It is the only protein we identified in secreted *Brugia* EV that is known to function specifically at the *Brugia*-host interface and its discovery in EV provides an additional mechanism for delivery of this protein to host tissues: EV proteins are generally not identified in secretome profiles so the presence of BmMIF-1 in both the secretome [13,71] and the EV proteome suggests it is both freely secreted and packaged in EV. Other EV proteins have described immunomodulatory properties in other systems, supporting the hypothesis that parasite-derived EVs function at the host-parasite interface. For example, galectin-1 and -2 were amongst the most abundant EV proteins. Galectins are glycan-binding proteins with well-established and complex roles in immune response modulation. Best understood is the ability of host galectins to bind glycans on the surface of T cells to regulate T cell signaling, activation profile, survival and cytokine production (reviewed in [74]). Within the context of a wider Th2 environment of the type established during parasite infection, galectin-1 and -2 have been shown to inhibit Th1 cytokine release from T cells [75,76] and promote Th2 cytokine release [77,78]. Relevant to the current work, there is evidence galectin-1 elicits alternative activation phenotypes in macrophages [79] and may facilitate parasite invasion; galectin-1 inhibited IL-12 production in *Trypanosoma cruzi*-infected macrophages and increased parasite replication [80]. Whilst there is strong evidence for host galectin-1 and -2 as contributors to a Th2 type environment and alternative activation of macrophages, the role for parasite-derived galectins in driving this response has not been examined but is undoubtedly intriguing.

Of interest in the proteomic data was the disparate complexity of male and female EV proteomes; 74 proteins were identified in the female sample whereas only 20 were identified in the male sample. This is consistent with the wider *Brugia* secretome, where female parasites secrete more proteins and in greater amounts than male worms [13,14] Further, we noted that functional proteins, such as those with putative immunomodulatory roles were enriched in the female profile (amongst others, BmMIF-1 was found in female EV but not male EV). Again, this has previously been suggested in discussions of the *Brugia* secretome [14] and the work presented here supports this paradigm and extends it to EV. One factor that may account for this diverged EV cargo is where the EV are generated and from where they are released. Although our data in mf suggests the ES pore may be an important site for EV release in that life stage, it might be expected that reproductive structures may be important anatomical routes for EV release in adults. Thus it might be expected that EV released via female reproductive structures would have qualitative and quantitative differences in EV cargo from those released by male reproductive structures. It is clear the site of vesicle generation will impact cargo and this underscores the importance of identifying validated markers of parasite EV such that vesicle genesis and release throughout the life cycle can be defined. As to the role of these EV at the host-parasite interface, the reduction of immunomodulatory proteins in male EVs suggests vesicle release may serve sexually dimorphic functions; female EVs may be more effective at host immunomodulation than male vesicles. It is not clear why male and female parasites would have discrepant functional roles in maintaining infection but a reduced immunomodulatory capability in males may simply be a result of redundancy; male and female worms associate closely in infected lymphatic vasculature and it might be expected that males would benefit from host immunomodulation driven by females.

The therapeutic effects of ivermectin against filarial nematodes are ambiguous. Chronic inhibition of embryogenesis and fertility of adult worms [81–85] may contribute but do not explain the acute clearance of larval stages from the blood. A novel mode of action was proposed by Moreno et al [56] who suggested the microfilaricidal action of IVM may include inhibition of protein release from the mf excretory pore. Our experiments exploring the effect of ivermectin on parasite EV release show that IVM rapidly (within 24 hrs) inhibits EV release directly or indirectly from all life stages of *B*. *malayi* examined. This phenotype is also observed in *D*. *immitis* L3 but not in a specific field isolate which demonstrates a reduced sensitivity to IVM [58]. These data are significant because they link the therapeutic efficacy of IVM to EV release by the parasite and support the hypothesis that the therapeutic effects of IVM against filarial nematodes may be explained, at least in part, by the inhibition of parasite EV release. This is consistent with, and complementary to, the findings of Moreno et al [56] but the data here is more explicit and extends this to other life stages. In this manuscript and elsewhere [32] we have shown that *Brugia* EVs contain putative immunomodulatory effectors, both proteins and small RNA species, and that IVM inhibits EV release by larval and adult life stages. It is possible that by preventing the release of these EV-bound effectors, the ability of the parasite to manipulate or evade the host response is impaired, allowing the host immune response to drive rapid parasite clearance. In support of this overall hypothesis, it will be important to not only titrate down the IVM concentration used and confirm the drug effect at therapeutically relevant concentrations but to determine if the effect extends to other members of the macrocyclic lactones. In addition to shedding light on IVM mechanisms of action, the work presented here may have implications for how we develop new anti-filarial drugs. If one IVM mechanism of action is to inhibit EV release from filarial nematodes then it stands that this new phenotype may be leveraged in screening platforms for drug discovery efforts. The output has advantages, it may well be a better indicator of anti-filarial activity than simply worm motility or viability, and its assessment is objectively quantifiable but several hurdles must be overcome. For one, it is highly worm intensive and significant numbers of parasites are required to meet the demands of current EV isolation methods. Also, EV isolation is time consuming and not well suited to high-throughput platforms. Alternative faster and more sensitive assays that indirectly quantify EV release via protein or small RNA markers may provide a solution. The work presented here, therefore, not only provides insight into an explicit immunomodulatory mechanism employed by filarial nematode parasites but also refines a potential explanation for ivermectin’s mechanism of action against filarial nematodes and identifies a new phenotype that may help identify a next generation of anti-filarial drugs.

## Supporting information

S1 Table *B*. *malayi* adult male EV proteome. Complete list of proteins identified in adult male *Brugia malayi* extracellular vesicle (EV) proteome. Proteins extracted from EVs were subject to nano-scale LC-MS/MS. Spectral data were searched against the *B*. *malayi*, *Ascaris suum* and *Caenorhabditis elegans* UniProt databases using MASCOT to identify proteins. The proteins and the name of the gene encoding that protein (GN) is listed. UniProtKB (www.uniprot.org) identifiers are provided for each protein along with molecular weight (kDa). Proteins are ranked by their normalized spectral abundance factor (NSAF), a simple indicator of protein abundance in the sample reflecting the effect of protein length. *proteins identified in separate proteomic runs such that NSAF can not be compared.
(XLSX)

S2 Table *B*. *malayi* adult female EV proteome. Complete list of proteins identified in adult female *Brugia malayi* extracellular vesicle (EV) proteome. Proteins extracted from EVs were subject to nano-scale LC-MS/MS. Spectral data were searched against the *B*. *malayi*, *Ascaris suum* and *Caenorhabditis elegans* UniProt databases using MASCOT to identify proteins. The proteins and the name of the gene encoding that protein (GN) is listed. UniProtKB (www.uniprot.org) identifiers are provided for each protein along with molecular weight (kDa). Proteins are ranked by their normalized spectral abundance factor (NSAF), a simple indicator of protein abundance in the sample reflecting the effect of protein length.
(XLSX)

S1 Fig There is no preferential internalization of *B*. *malayi* life stage EVs. 5 x 10^5^ J774A.1 macrophages were incubated with 1 x 10^7^ PKH67-labeled microfilarial (mf), L3, L4, adult male (AM) or female (AF) EVs for 24hr. Media was removed, cells washed and fluorescence quantified using flow cytometry. There was no significant difference in internalization of the different life stage EVs. N = 5 (except L4 N = 2), mean ± SEM, P>0.05.
(TIFF)

S2 Fig Gene ontology (GO) mapping of annotated adult male EV proteome by cellular component. Visualization of annotated adult male EV proteome using GO terms associated with cellular components. Each node includes the following information: GO ID, the name of the GO ID, the nodescore (an evaluation of the number of sequences converging at the node and the distance to the node where each sequence was annotated) and the number of sequences assigned that GO term. The most relevant nodes are highlighted by increased color intensity.
(TIFF)

S3 Fig Gene ontology (GO) mapping of annotated adult female EV proteome by cellular component. Visualization of annotated adult female EV proteome using GO terms associated with cellular components. Each node includes the following information: GO ID, the name of the GO ID, the nodescore (an evaluation of the number of sequences converging at the node and the distance to the node where each sequence was annotated) and the number of sequences assigned that GO term. The most relevant nodes are highlighted by increased color intensity.
(TIFF)

S4 Fig Gene ontology (GO) mapping of annotated adult male EV proteome by molecular function. Visualization of annotated adult male EV proteome using GO terms associated with molecular function. Each node includes the following information: GO ID, the name of the GO ID, the nodescore (an evaluation of the number of sequences converging at the node and the distance to the node where each sequence was annotated) and the number of sequences assigned that GO term. The most relevant nodes are highlighted by increased color intensity.
(TIFF)

S5 Fig Gene ontology (GO) mapping of annotated adult female EV proteome by molecular function. Visualization of annotated adult female EV proteome using GO terms associated with molecular function. Each node includes the following information: GO ID, the name of the GO ID, the nodescore (an evaluation of the number of sequences converging at the node and the distance to the node where each sequence was annotated) and the number of sequences assigned that GO term. The most relevant nodes are highlighted by increased color intensity.
(TIFF)
