# Supplementary figures and images for: Profiling extracellular vesicle release by the filarial nematode Brugia malayi reveals sex-specific differences in cargo and a sensitivity to ivermectin
Source: PLoS Negl Trop Dis. 2018 Apr 16;12(4):e0006438. doi: 10.1371/journal.pntd.0006438 (PMC5919703; doi:10.1371/journal.pntd.0006438)

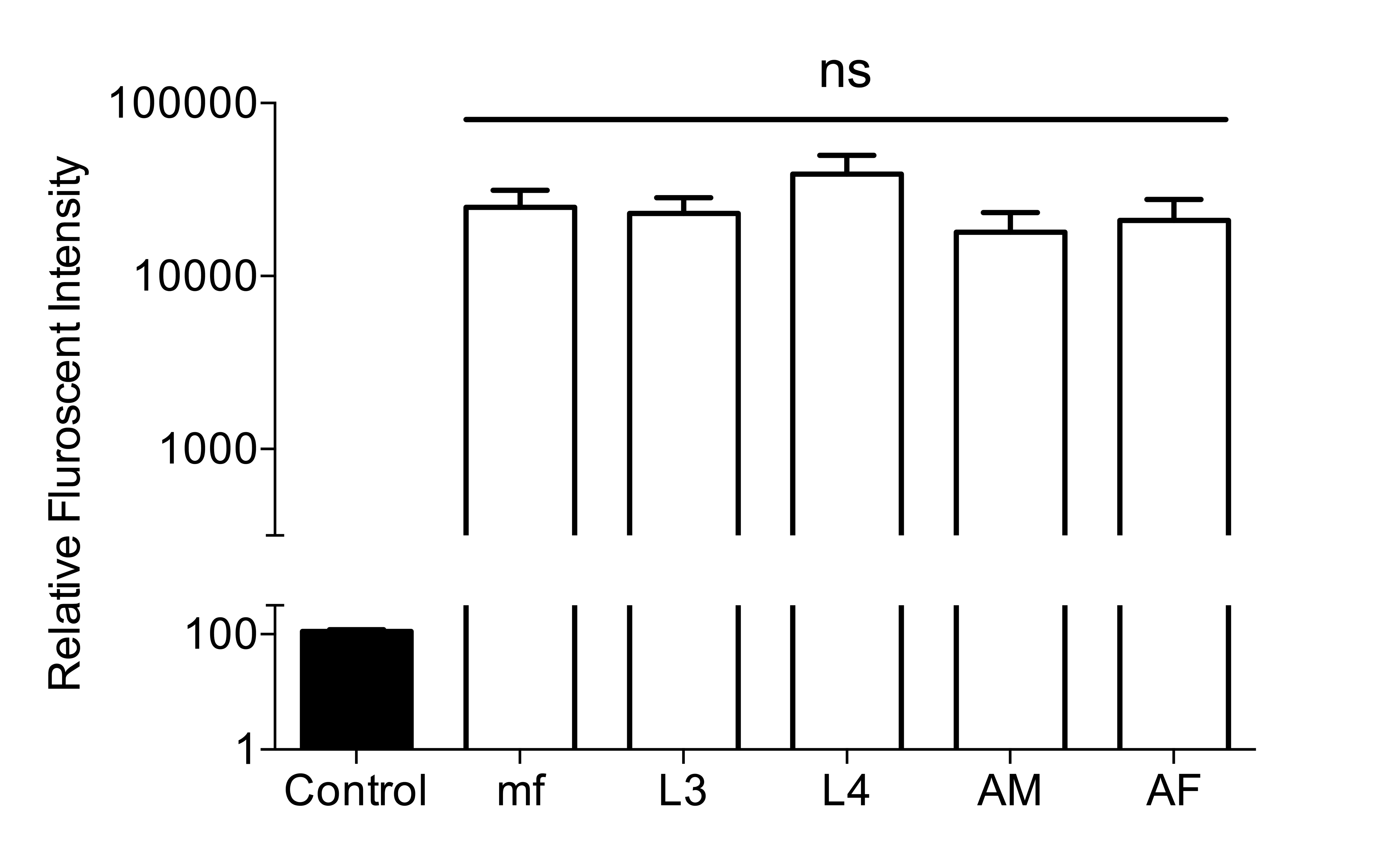

Supplement: S1 Fig — 5 x 10^5^ J774A.1 macrophages were incubated with 1 x 10^7^ PKH67-labeled microfilarial (mf), L3, L4, adult male (AM) or female (AF) EVs for 24hr. Media was removed, cells washed and fluorescence quantified using flow cytometry. There was no significant difference in internalization of the different life stage EVs. N = 5 (except L4 N = 2), mean ± SEM, P>0.05. (TIFF) [file pntd.0006438.s003.tiff]

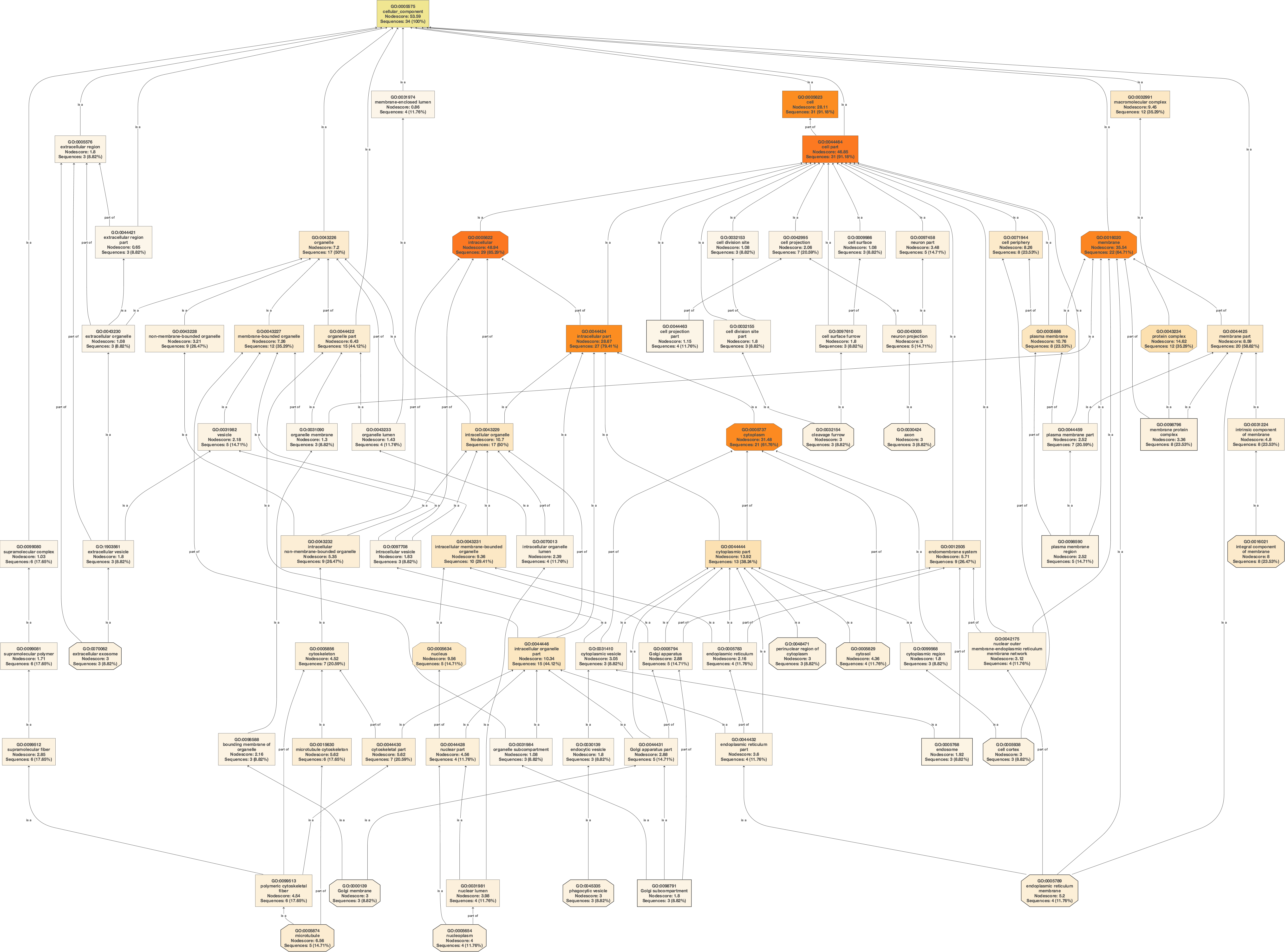

Supplement: S2 Fig — Visualization of annotated adult male EV proteome using GO terms associated with cellular components. Each node includes the following information: GO ID, the name of the GO ID, the nodescore (an evaluation of the number of sequences converging at the node and the distance to the node where each sequence was annotated) and the number of sequences assigned that GO term. The most relevant nodes are highlighted by increased color intensity. (TIFF) [file pntd.0006438.s004.tiff]

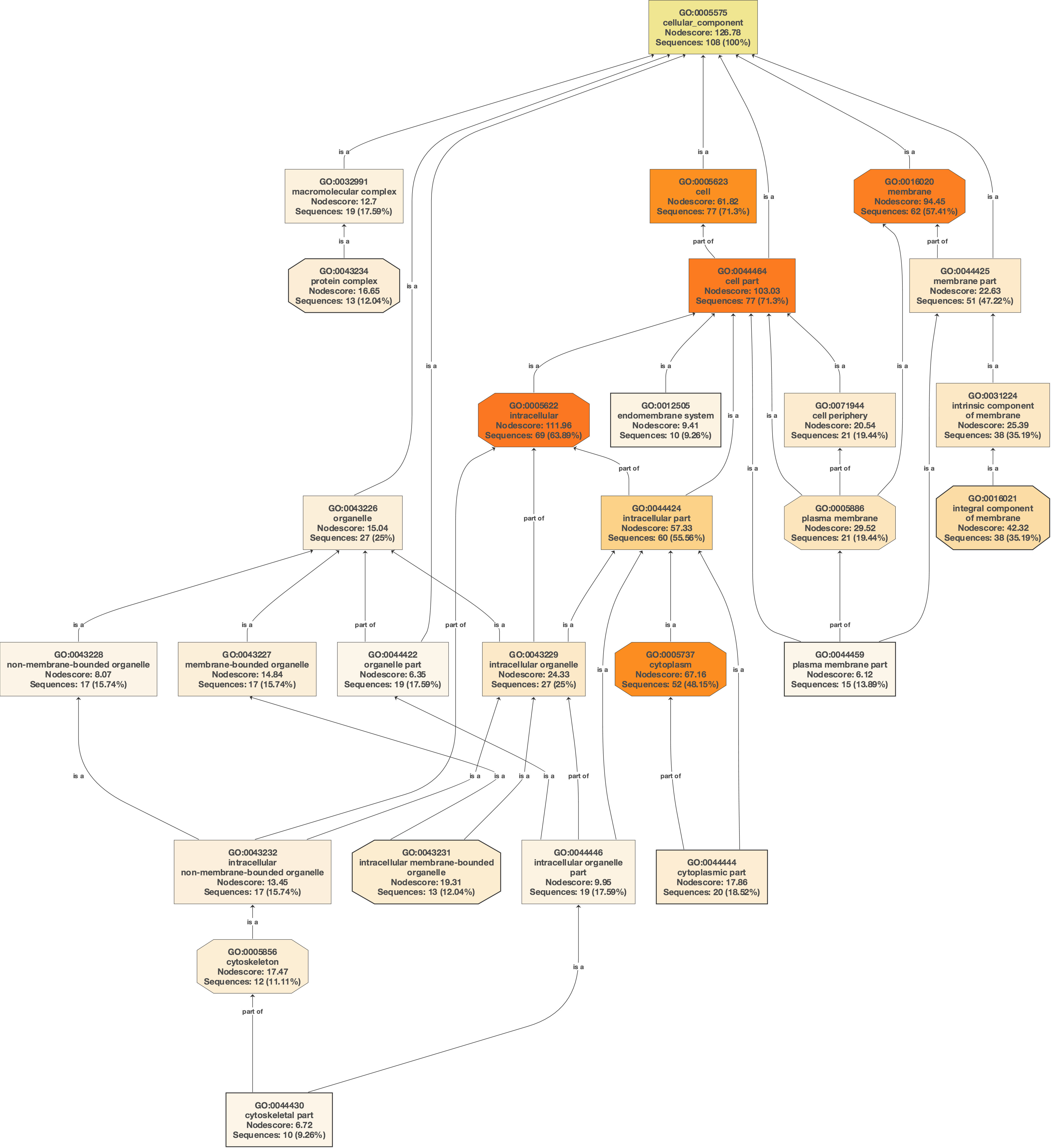

Supplement: S3 Fig — Visualization of annotated adult female EV proteome using GO terms associated with cellular components. Each node includes the following information: GO ID, the name of the GO ID, the nodescore (an evaluation of the number of sequences converging at the node and the distance to the node where each sequence was annotated) and the number of sequences assigned that GO term. The most relevant nodes are highlighted by increased color intensity. (TIFF) [file pntd.0006438.s005.tiff]

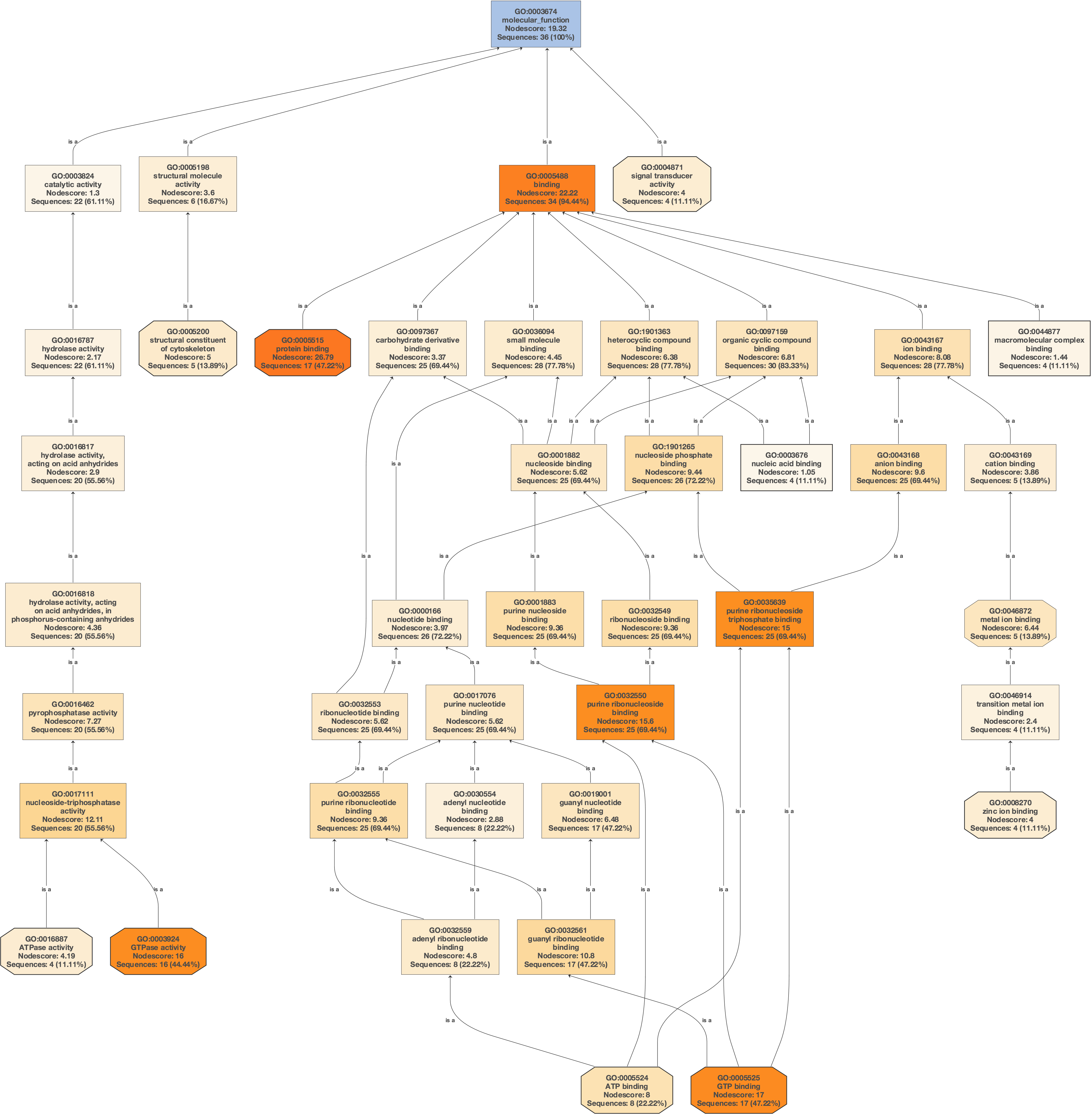

Supplement: S4 Fig — Visualization of annotated adult male EV proteome using GO terms associated with molecular function. Each node includes the following information: GO ID, the name of the GO ID, the nodescore (an evaluation of the number of sequences converging at the node and the distance to the node where each sequence was annotated) and the number of sequences assigned that GO term. The most relevant nodes are highlighted by increased color intensity. (TIFF) [file pntd.0006438.s006.tiff]

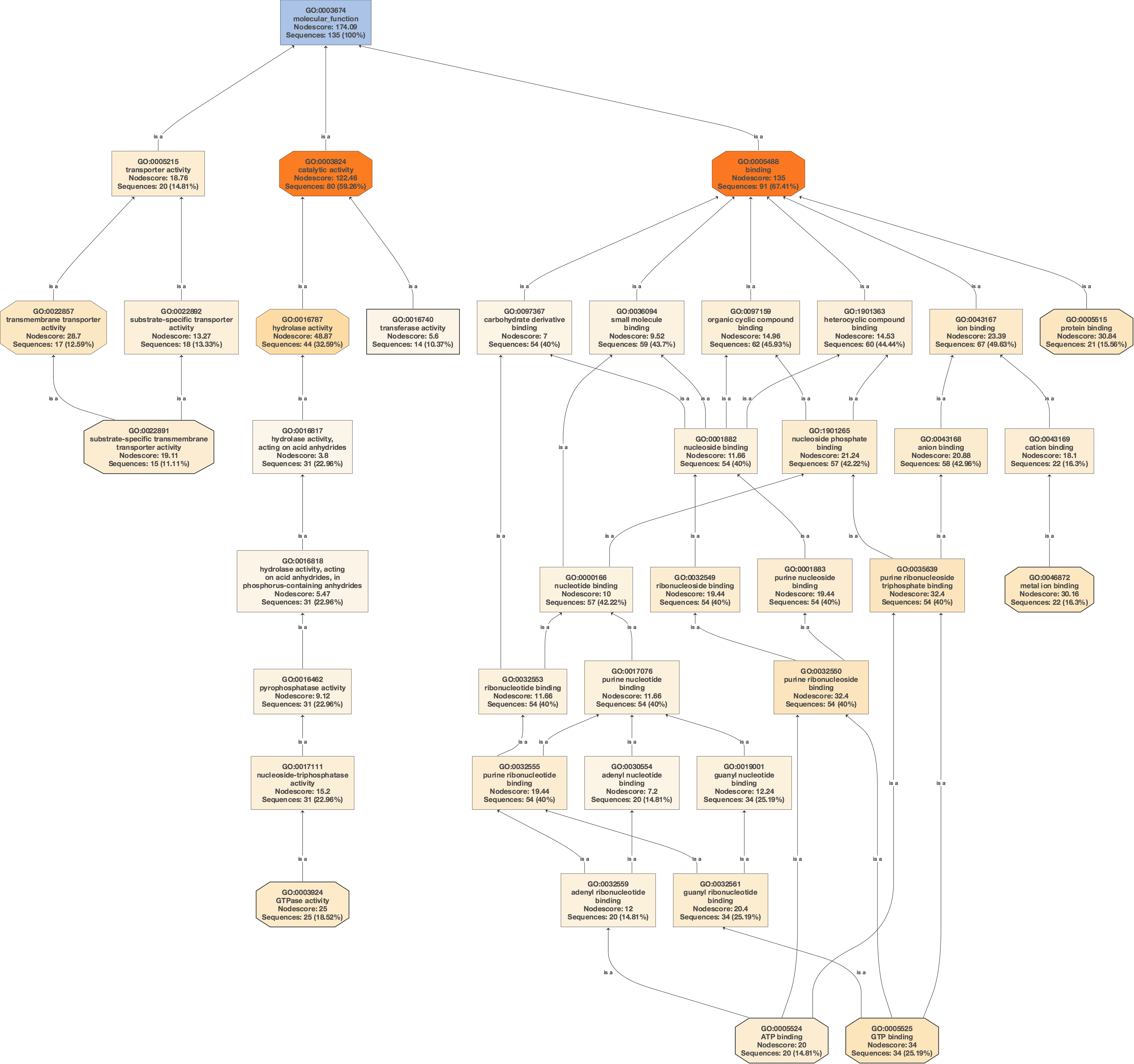

Supplement: S5 Fig — Visualization of annotated adult female EV proteome using GO terms associated with molecular function. Each node includes the following information: GO ID, the name of the GO ID, the nodescore (an evaluation of the number of sequences converging at the node and the distance to the node where each sequence was annotated) and the number of sequences assigned that GO term. The most relevant nodes are highlighted by increased color intensity. (TIFF) [file pntd.0006438.s007.tiff]
